# Low Rates of Pointing in 18-Month-Olds at Risk for Autism Spectrum Disorder and Extremely Preterm Infants: A Common Index of Language Delay?

**DOI:** 10.3389/fpsyg.2019.02131

**Published:** 2019-10-09

**Authors:** Alessandra Sansavini, Annalisa Guarini, Mariagrazia Zuccarini, Jessica Zong Lee, Giacomo Faldella, Jana Marie Iverson

**Affiliations:** ^1^Department of Psychology, University of Bologna, Bologna, Italy; ^2^Department of Psychology, University of Pittsburgh, Pittsburgh, PA, United States; ^3^Department of Medical and Surgical Sciences, University of Bologna, Bologna, Italy

**Keywords:** gestures, pointing, early indexes of language delay, infants at risk for ASD, extremely preterm infants

## Abstract

Infants with an older sibling with an Autism Spectrum Disorder diagnosis (Sibs ASD) are at high risk for language delay (LD) as well as infants born preterm, especially those with an extremely low gestational age (ELGA, GA ≤ 28 weeks). Gestures play a crucial role in language development and delays in gesture production may have negative cascading effects on it. The present exploratory study examined gesture production in 18-month-old infants with different underlying risks for LD. Seventy monolingual United States infants (41 Sibs ASD with no eventual ASD diagnosis and 29 infants with a typically developing older sibling -Sibs TD) and 40 monolingual Italian infants (20 ELGA without major cerebral damages, congenital malformations or sensory impairments and 20 full-term - FT infants, GA ≥ 37 weeks) were included. Both groups were followed longitudinally from 18 to 24, 30, and 36 months (corrected for ELGA infants). A 30-minute mother-infant play session with age-appropriate toys was video recorded at 18 months of age. Deictic (requesting, pointing, showing and giving), conventional, and representational gestures spontaneously produced by infants were coded; rate per 10 min was calculated. LD was defined as a score ≤10th percentile on the American English or Italian version of the MacArthur-Bates CDI on at least two time points between 18 and 36 months. Fifteen Sibs ASD and 9 ELGA infants were identified as infants with LD. Sibs ASD-LD and Sibs ASD-no LD produced fewer pointing gestures compared to Sibs TD (*p* = 0.038; *p* = 0.004); ELGA-LD infants produced significantly fewer pointing gestures than ELGA-no LD (*p* = 0.024) and FT (*p* = 0.006) infants. Low rates of pointing at 18 months are a marker of LD in Sibs ASD and ELGA infants. The potential implications of reduced pointing production and characteristics of different populations at risk for LD should be considered for understanding the emergence of LD.

## Introduction

Language development is considered a reliable indicator of development and is related to later school achievements (Nelson et al., 2006). Language delay (LD) can be identified between 18 and 36 months in young children with limited expressive vocabularies, equivalent to the 10th percentile or below compared to normative values, and who are free from cognitive, neurological, socio-emotional, or sensory deficits (Rescorla, 2011). The prevalence of LD in large population-based cohorts ranges from 13 to 20% for 2-year-old children (Zubrick et al., 2007) and from 5 to 12% for children 2 to 5 years old (Law et al., 2000). Among children with LD, the majority develops language skills in the average range by 3–4 years onward, although maintaining lower scores than their peers, but some persist with LD that will affect later school achievements (Rescorla, 2011). Early identification of children at risk for language delay (LD) is thus important for intervention and improvement.

The risk of exhibiting LD is greater in populations characterized by perinatal risk factors (infants born preterm; Law et al., 2000; Nelson et al., 2006) or by genetic factors (younger siblings of children with autism spectrum disorder; ASD; e.g., Ozonoff et al., 2014). These two at-risk populations are characterized by high interindividual variability, with about 30–40% of infants developing a LD (Ozonoff et al., 2014; Sansavini et al., 2014; Iverson et al., 2018). Thus, it is important to examine whether early communicative indices exist in these populations that can identify infants with LD. The present exploratory study intends to address this issue focusing on these two populations characterized by different underlying biological and environmental risks for LD, i.e., infants who have an older sibling with ASD (Sibs ASD) and infants born extremely preterm (extremely low gestational age- ELGA), compared to typically developing (TD) comparison groups, i.e., infants with a typically developing older sibling and no family history of ASD (Sibs TD) and full-term infants (FT), respectively. Finding a common communicative index of LD that can be utilized at 18 months across different populations can increase our understanding of typical and atypical language development and permit early identification of infants with LD and, consequently, early planning of customized interventions. In addition, analyzing the emergence of LD in two populations with different underlying biological and environmental risks can shed light on different phenotypes of LD.

### Populations at Risk for LD: Sibs ASD and ELGA Infants

Sibling with ASD are known to be at heightened risk for ASD (18.9% ASD recurrence rate; Ozonoff et al., 2011). Studies employing semistructured assessments in the lab or naturalistic observations at home have also revealed that Sibs ASD (even those who do not receive an ASD diagnosis) show slower communicative-linguistic development compared to infants who have a typically developing (TD) older sibling and no family history of ASD (Sibs TD) across the first 2 years of life (Franchini et al., 2018). Indeed, toward the end of the first year, TD infants start guiding adult’s attention by alternating their gaze between the adult’s face and objects that have caught their attention (alternating gaze behavior) and, subsequently, also using pointing or vocalizing. These initiating joint attention behaviors create opportunities for communication, triggering adults’ attending to infant behavior and labeling the objects of attention, that support infants’ language development (Cochet and Byrne, 2016). Recent research has reported that Sibs ASD engage less in interactive behaviors that can potentially initiate joint attention. Specifically, they engage less in alternating gaze during interaction with an adult at 10 months (Thorup et al., 2018), show lower rates of behavioral requesting using gaze, pointing and giving gestures at 12 months, initiate joint attention with alternated gaze and pointing less frequently at 15 months (Cassel et al., 2007), and produce fewer deictic gestures, especially pointing, at 13–14 and 18 months (Yirmiya et al., 2006; Winder et al., 2013; Leezenbaum et al., 2014; Thorup et al., 2018). In addition, they exhibit lower rates of vocalizations and words at 13 and 18 months (Winder et al., 2013) and slower acquisition of vocabulary between 9 and 24 months (Franchini et al., 2018).

Recent studies have more specifically investigated interindividual differences among Sibs ASD who did not receive an ASD diagnosis, reporting relatively high rates of LD, with 35 to 40% of them meeting criteria for LD between the ages of 2–3 years (Landa and Garrett-Mayer, 2006; Yirmiya et al., 2006; Parladé and Iverson, 2015; LeBarton and Iverson, 2016; Iverson et al., 2018). For instance, a study has shown that Sibs ASD who did not receive an ASD diagnosis but were identified with LD at 36 months of age (Sibs ASD-LD) demonstrated smaller acceleration in growth trajectories of early and later gestures and of word comprehension and production already from 8 months of age with respect to Sibs ASD who did not receive an ASD diagnosis and had no LD (Sibs ASD-no LD) and to Sibs TD. Although differences in gestures and vocabulary acquisition among Sibs ASD-LD, Sibs ASD-no LD and Sibs TD tended to decrease during the second year, a much slower rate of growth for Sibs ASD-LD continued to be observed (Iverson et al., 2018). Further studies have shown that, between 14 and 24 months of age, Sibs ASD-LD tended to engage in initiating joint interaction behaviors, such as alternated gaze, pointing and vocalizations, less frequently, compared to Sibs ASD-no LD (Heymann et al., 2018) and at 24 months they exhibited less pointing than Sibs ASD-no LD (LeBarton and Iverson, 2016). These findings suggest the need to understand potential early indices of LD in Sibs ASD, and that differences in gesture production, particularly in pointing, might provide early indications of risk of later LD in this population.

Preterm birth, i.e., a birth occurring before 37 weeks of gestational age- GA- (March of Dimes et al., 2012), is also a risk factor for LD (Sansavini et al., 2010b, 2014). Several studies have shown that multiple atypical biological constraints and environmental conditions characterizing preterm birth lead to atypical developmental trajectories in several domains (Sansavini et al., 2011a). Among them, language is particularly vulnerable up to adolescence (e.g., Barre et al., 2011; Sansavini et al., 2011a; Van Noort-Van Der Spek et al., 2012; Sansavini et al., 2014; Guarini et al., 2016). A wide heterogeneity has also been shown within the preterm population due to interactions among level of neonatal immaturity, medical complications, and environmental and social characteristics (Sansavini et al., 2011b). Indeed, infants born before 28 weeks of GA (i.e., extremely low gestational age-ELGA- infants) are at higher risk for multiple developmental difficulties and impairments, even in the absence of major cerebral damage (Marlow et al., 2005; Anderson and Doyle, 2008; de Kievet et al., 2009; Barre et al., 2011; Sansavini et al., 2011c, 2014; Van Noort-Van Der Spek et al., 2012). However, only a few studies have specifically focused on communicative and linguistic skills in ELGA infants. At 24 months of corrected age, compared both to FT and VLGA infants (i.e., very low gestational age- ≤32 weeks), ELGA infants were reported by their parents to produce fewer words on the MacArthur-Bates Communicative Development Inventory- CDI (Foster-Cohen et al., 2007). They also exhibited lower scores in receptive and expressive language, directly examined with standardized tests, in comparison to FT infants at several points of assessment between 12 and 36 months corrected age (Sansavini et al., 2014, 2015a). Furthermore, a recent study observing infant communication skills in mother-infant interaction sessions at 12 months (corrected age for ELGA infants) found that ELGA dyads, compared to FT dyads, were characterized by less frequent symmetric co-regulation patterns, defined by a shared focus of attention and reciprocal active participation and innovation during interaction (Sansavini et al., 2015b). In addition, another study conducted with the same context of observation, showed that ELGA infants produced fewer pointing, giving, and representational gestures than FT peers (Benassi et al., 2016).

Concerning relations between early gesture production and later language development in the preterm population, two studies of VLGA infants reported that gesture production together with word comprehension and word production on the CDI at 18 months were predictive of word production at 24 months (Sansavini et al., 2011b); and gestures between 9 and 13 months of age were positively related to language skills at 5 years of age (Stolt et al., 2016). These findings support the hypothesis that gestures are relevant precursors of language development (Iverson and Goldin-Meadow, 2005; Bavin et al., 2008; Capirci and Volterra, 2008). However, to date, no study has explored these associations specifically in ELGA infants. This is surprising, since ELGA infants are at high risk for LD between 2 and 3 years of age (Sansavini et al., 2014; Vohr, 2014). Therefore, exploring early gesture development in ELGA infants might be informative of potential indicators of later LD.

### Delay in Gesture Production as Early Index of LD

A large body of work has described developmental continuity from gestures to words (e.g., see Volterra et al., 2017 for a review) with consistent evidence of tight developmental relations between gestures and language (Iverson and Thelen, 1999; Bates and Dick, 2002) and of a crucial role for pointing gesture in early language development (e.g., see Colonnesi et al., 2010 for a meta-analysis).

By gesturing, children can obtain and maintain attention of the adult, thereby establishing new language learning opportunities (Bates et al., 1979; Capone and McGregor, 2004). In particular, pointing, emerging in TD infants between 10 and 12 months and becoming frequent and consolidated between 12 and 18 months (Fenson et al., 2007; Caselli et al., 2015; Lüke et al., 2017), elicits object labeling from caregivers (Goldin-Meadow et al., 2007), triggering social and verbal exchanges that support language acquisition. Since pointing is often produced together with gaze alternation to the recipient, delays in pointing may reduce infant-initiated joint interactions with caregivers, which may in turn alter input to the infant (Leezenbaum et al., 2014). Indeed, both deictic and representational gestures have been shown to be strongly associated with word comprehension (Sansavini et al., 2010a; Caselli et al., 2012) and word production (Capirci et al., 1996; Sansavini et al., 2010a). In particular, pointing at 12 and 18 months predicts later vocabulary (Camaioni et al., 1991; Iverson and Goldin-Meadow, 2005) and sentence complexity (Rowe and Goldin-Meadow, 2009). In addition, using less pointing between 12 and 18 months resulted associated with later LD in infants with an older sibling with LD (Lüke et al., 2017) and in infants with pre- or perinatal unilateral brain lesions (Sauer et al., 2010).

The above findings suggest that the study of gesture development may serve both as an indicator of infants’ communicative-linguistic level and as a tool to identify children at risk for LD (Thal and Tobias, 1992; Crais et al., 2009; Goldin-Meadow, 2015). However, it is still unclear whether common indexes of LD, such as frequency of gesture production and production rate of specific types of gestures, like pointing, may be early indicators of enhanced LD risk for infants from different and heterogeneous populations, such as Sibs ASD and ELGA infants, who will eventually exhibit LD.

### Current Study

As suggested by D’Souza et al. (2016), comparisons between a clinical/at risk population and a TD group will show whether the developmental pattern of that clinical/at risk population can be considered typical or atypical. In addition, comparisons among clinical/at risk populations will contribute to understanding whether a developmental pattern is specific to a certain clinical/at risk population or is a common sign of a developmental deficit across several populations. Thus, cross-population studies from early stages of development, when delays and disorders start to emerge, could shed light on their phenotypes. The present exploratory study was therefore designed to examine potential differences in gesture production at 18 months among subgroups of infants from different populations at risk for LD and TD comparison infants.

Specifically, this research examined gesture production in 18-month-old infants from two populations characterized by different underlying biological and environmental risks for LD: infants who have an older sibling with ASD (Sibs ASD) with no eventual ASD diagnosis and infants born extremely preterm (extremely low gestational age- ELGA) without major cerebral damages, congenital malformations or sensory impairments. Both populations are characterized by wide interindividual variability, with some infants exhibiting LD and others not. To this end, each group at risk for LD was further classified into two subgroups according to the presence or absence of LD (LD vs. no LD), ascertained on at least two time points between 18 and 36 months.

Two groups of typically developing (TD) infants were also included. Sibs ASD with no language delay (Sibs ASD-no LD) and with language delay (Sibs ASD-LD) were compared to infants with a TD older sibling and no family history of ASD (Sibs TD). ELGA infants with no language delay (ELGA-no LD) and with language delay (ELGA-LD) were compared to a group of full-term infants (FT). We expected to observe lower rates of gestures, particularly pointing, at 18 months in both Sibs ASD-LD and ELGA-LD infants, relative to their comparison groups (Sibs TD and FT infants, respectively) as well as to their peers without LD (Sibs ASD-no LD and ELGA-no LD, respectively). By contrast, no significant differences in gesture production at 18 months were expected between Sibs ASD-no LD and ELGA-no LD and their respective comparison groups.

## Materials and Methods

### Participants

Two groups of infants at risk for language delay (LD) and their respective comparison groups were included in this study. Infants in both groups were part of larger longitudinal studies monitoring their development from birth up to 3 years of age. The first group at risk for LD consisted of 41 monolingual United States infants (23 females) who had an older sibling with an ASD diagnosis (Sibs ASD) but no eventual ASD diagnosis. The older sibling’s ASD diagnosis was independently confirmed prior to the infant’s study enrollment via administration of the Autism Diagnostic Observation Schedule (ADOS; Lord et al., 2000). The older sibling had to score above the threshold for Autism on the ADOS and receive clinical judgment of Autism based on DSM-IV criteria. The Sibs ASD group was compared to a group of 29 monolingual United States infants (16 females) (Sibs TD), who had a typically developing older sibling and no family history of ASD. All infants were recruited through a university Autism Research Program, parent support organizations, and local agencies and schools serving families of children with ASD. All infants were born at term from uncomplicated pregnancies and had no visual or hearing impairments. Socio-demographic characteristics of the Sibs ASD and Sibs TD are presented in Table 1. The Sibs ASD and Sibs TD were comparable on gender, maternal education level, and maternal age.

**TABLE 1:** Socio-demographic characteristics of the Sibs ASD, Sibs TD, ELGA and FT groups.

		**Sibs ASD (*n* = 41)**	**Sibs TD (*n* = 29)**	**ELGA (*n* = 20)**	**FT (*n* = 20)**
Infant gender	Female, *n* (%)	23 (56)	16 (55)	11 (55)	8 (40)
Maternal education level	Middle-Middle/High-school *n* (%)	11 (27)	4 (14)	12 (60)	9 (45)
	High-College/Higher degree *n* (%)	30 (73)	25 (86)	8 (40)	11 (55)
Maternal Age	*Mean (SD) range*	33.9 (4.5) 23–45	31.9 (4.6) 24–42	36.2 (4.8) 27–44	34.8 (3.0) 30–41

The second group at risk for LD consisted of 20 Italian monolingual preterm infants with an extremely low gestational age (ELGA, GA ≤ 28 weeks, 11 females). Gestational age was defined according to the date of the mother’s last menstrual period and confirmed by first-trimester early ultrasonography. The ELGA infants had a mean gestational age of 25.8 weeks (*SD* = 1.5) and a mean birth weight (BW) of 803 g (*SD* = 191). The ELGA group was compared to a control sample of 20 Italian full-term Italian monolingual infants (FT, 8 females), with a GA ≥ 37 weeks. The mean gestational age of FT infants was 39.4 weeks (*SD* = 1) and their mean birth weight was 3392 g (*SD* = 448). All ELGA infants were born at the Neonatal Intensive Care Unit (NICU) of the Hospital of the University of Bologna; the FT infants were recruited from the same hospital. All infants came from Italian-speaking households. The inclusion criteria for the ELGA and FT groups were absence of major cerebral damages, congenital malformations and visual or hearing impairments. Socio-demographic characteristics of the ELGA and FT groups are displayed in Table 1. The ELGA and FT infants were comparable on gender, maternal education level, and maternal age (For description of ELGA infants’ biological and medical characteristics, see Supplementary File).

### Measures

Parents of Sibs ASD and Sibs TD completed the American English version of the MacArthur-Bates Communicative Development Inventory (CDI)- Words and Sentences (WS) long form (CDI-WS, Fenson et al., 2007) at infant ages 18 and 24 months and the CDI-III (Fenson et al., 2007) at 36 months. Parents of the ELGA and FT groups completed the Italian version of the CDI-WS long form (Primo Vocabolario del Bambino: gesti, Parole e Frasi, PVB-PF, Caselli et al., 2015) at 18, 24, 30, and 36 months of infant’s age (corrected for weeks of prematurity). The CDI-WS is a valid and reliable tool used across different languages and cultures in research and clinical contexts to assess vocabulary production and identify LD in TD infants (Fenson et al., 2007; Caselli et al., 2015), infants at risk for ASD (Iverson et al., 2018), and infants born preterm (Sansavini et al., 2010b, 2011b; Stolt et al., 2016). The CDI-WS is normed for children between 16 and 30-month-old in the American English version (Fenson et al., 2007) and for children between 18 and 36 months in the Italian version (Caselli et al., 2015). It consists of 680 words organized into 22 semantic categories in the English version and of 670 words organized into 23 semantic categories in the Italian version. The CDI-III is an extension of the American English version of the CDI-WS normed for children between 30 and 37 months of age (Fenson et al., 2007) and consists of 100 words. The parent is asked to check the words his/her child spontaneously produces; a score of 1 is given for each item checked. The total number of words produced is computed. Both American English and Italian CDI versions include also a section on children’s use of morphology and syntax that was not considered for the purpose of the present study.

At 36 months, children in the Sibs ASD group were administered the ADOS-G (Lord et al., 2000) and the Mullen Scales of Early Learning (MSEL; Mullen, 1995). The ADOS-G is a structured play schedule that provides systematic probes for symptoms of ASD in social interaction, communication, play, and repetitive behaviors, and has standard administration and scoring schema. All of the Sibs ASD in this study scored below the threshold for ASD and none received an ASD diagnosis. The MSEL is a developmental assessment of language, cognitive, and motor functioning from birth to 68 months, standardized for the United States population. It is organized into five subscales: gross motor, fine motor, visual reception (or non-verbal problem solving), receptive language, and expressive language. Each subscale is standardized to calculate standard score, percentile and age-equivalent score. The receptive and expressive language subscale scores, along with the CDI-III scores, were used to determine language outcomes.

### Outcome Classification

Language delay was defined as a score ≤10th percentile on the American English or Italian version of the CDI on at least two time points between 18 and 36 months. On the American English version of the CDI-WS long form (CDI-II, Fenson et al., 2007), the 10th percentile corresponded to 16 words for males and 21 words for females at 18 months and to 63 and 92 words for males and females respectively at 24 months; on the CDI-III (Fenson et al., 2007), the 10th percentile corresponded to 55 and 60 words for males and females respectively at 36 months. For the Italian version of the CDI-WS long form (PVB-PF, Caselli et al., 2015), the 10th percentile corresponded to 9 words at 18 months, 80 words at 24 months, 254 words at 30 months and 349 words at 36 months. Scores were not differentiated for gender in the PVB-PF.

Specifically, Sibs ASD infants were classified as language delayed (Sibs ASD-LD) if either of the following criteria were met (Parladé and Iverson, 2015; West et al., 2019; Iverson et al., 2018): a) standardized scores on the CDI-WS (Fenson et al., 2007) or CDI-III at or below the 10th percentile at more than one time point between 18 and 36 months (e.g., Heilmann et al., 2005); or b) standardized score on the CDI-III at or below the 10th percentile and a standardized score on the Receptive and/or Expressive Language subscales of the MSEL equal to or greater than 1.5 standard deviations below the mean at 36 months (e.g., Landa and Garrett-Mayer, 2006). Based on these criteria, 13 Sibs ASD were classified as LD (Sibs ASD-LD). The remaining 28 infants did not meet the criteria for LD (Sibs ASD-no LD). Sibs ASD-no LD and Sibs ASD-LD did not differ significantly on gender (males: Sibs ASD-LD = 62%; Sibs ASD-no LD = 32%; χ*^2^* = 3.16, *p* = 0.098), maternal education (high education level: Sibs ASD-LD: 54%; Sibs ASD-no LD = 82%; χ*^2^* = 3.62, *p* = 0.073) or maternal age (Sibs ASD-LD: *M* = 35; *SD* = 5.20; Sibs ASD-no LD = *M* = 33.32; *SD* = 4.10; *t* = 1.12; *p* = 0.27).

ELGA infants were classified as language delayed (ELGA-LD) if they had standardized scores on the PVB-PF (Caselli et al., 2015) at or below the 10th percentile at more than one time point between 18 and 36 months (Weismer and Evans, 2002). Using this criterion, 9 ELGA infants (6 males) were classified as LD (ELGA-LD). The remaining 11 ELGA infants were classified as not having LD (ELGA-no LD). ELGA-LD and ELGA-no LD children did not differ significantly on gender (males: ELGA-LD = 67%; ELGA-no LD = 27%; χ*^2^* = 3.10, *p* = 0.175), maternal education (high education level: ELGA-LD: 22%; ELGA-no LD = 55%; χ*^2^* = 2.15, *p* = 0.197) or maternal age (ELGA-LD: *M* = 36.22; *SD* = 5.61; ELGA-no LD: *M* = 36.18; *SD* = 4.36; *t* = 0.018; *p* = 0.99). With respect to biological and medical characteristics, ELGA-LD had a lower mean gestational age and they spent significantly more days in hospital compared to ELGA-no LD peers (ELGA-LD: GA weeks *M* = 24.9, *SD* = 1.3; days of hospitalization *M* = 113.4, *SD* = 28.5; ELGA-NO LD: GA weeks *M* = 26.5, *SD* = 1.2; days of hospitalization *M* = 73.5, *SD* = 22.9; see Supplementary File).

None of the Sibs TD or FT infants were classified as LD.

### Procedure

All infants were observed in a naturalistic play interaction session with their mother and age-appropriate toys at 18 months (corrected age for the ELGA infants). As in many studies investigating preterm infants’ development in the first 2 years of life, corrected age was used for ELGA infants in order to take into account their level of neuropsychological maturation (Sansavini et al., 2011a).

Mothers were asked to play with her infant as they normally would. For Sibs ASD and Sibs TD, the interaction was video-recorded for approximately 30–45 consecutive minutes at home. For ELGA and FT infants, the play session was video-recorded for approximately 30 consecutive minutes in a quiet room designed for observation at the day-hospital at the Unit of Neonatology of the Hospital of the University of Bologna. Ages at the 18-month observation were comparable across the four groups (Sibs ASD: *M* = 18.22 months; *SD* = 0.43; Sibs TD: *M* = 18.07; *SD* = 0.12; ELGA infants: *M* = 18.03; *SD* = 0.37; FT infants: *M* = 18.20; *SD* = 0.32).

All study procedures met the ethical guidelines for protection of human participants, including adherence to the legal requirements of the Country, and received a formal approval by the local Ethical Committees. The study from which Sibs ASD and Sibs TD were drawn was approved by the University of Pittsburgh Institutional Review Board. The ELGA and FT infant study was approved by the Ethical Committee of the Hospital of University of Bologna. The parents of all infants provided written informed consent for participation in the study, data analysis, and anonymized data publication.

### Coding

A trained observer, naïve to group membership, coded all communicative gestures spontaneously produced by the infants during the parent-infant play-interaction session at 18 months. Only gestures entailing an effort to direct caregiver’s attention were considered communicative (Iverson et al., 1994). Gestures were classified into one of three mutually exclusive gesture type categories: *deicti*c, *conventional* and *representational* (Capirci et al., 1996; Benassi et al., 2016). Among deictic gestures, *requesting/reaching* (extension of the arm with prone or supine open palm or repeated opening/closing of the hand with the aim to request something), *pointing* (articulation of the index finger directed toward a proximal or distal object with the aim to share attention or request), *showing* (holding up the object toward the partner while making eye contact), and *giving* (extension of the arm with the object in hand and directed toward the hand of another person) were coded; *conventional* gestures were ritualized or culturally defined; *representational* gestures had a specific referent and their primary semantic content did not change with context (the coding scheme has been published in Benassi et al., 2016).

The rate per 10 min for each gesture category was computed in order to take into account variation in session length across groups. Rates per 10 min were calculated by dividing raw frequencies of each category by the total session duration and then multiplying the result by 10.

### Reliability

To obtain intercoder reliability, additional trained coders naive to infant group membership independently coded a randomly selected 20% of the video-recorded sessions from each group.

For the Sibs ASD and Sibs TD groups, the intraclass correlation coefficient (ICC) for gesture production was 0.75. For the ELGA and FT groups, Cohen’s Kappa was calculated on gesture production obtaining a mean *K* value of 0.90. Values equal or higher than 0.75 are considered an index of excellent agreement both for ICC and Cohen’s Kappa (Cicchetti, 1994).

### Statistical Analyses

Statistical analyses were conducted using SPSS 23.0 for Windows with an alpha level of 0.05. Since data were not normally distributed, differences in gesture production (rates per 10 min) and word production among groups (Sibs TD, Sibs ASD-no LD, and Sibs ASD-LD; FT, ELGA-no LD, and ELGA-LD infants) were analyzed using the Kruskal-Wallis test. Pairwise comparisons were conducted using the Mann-Whitney test to further investigate differences between subgroups for Sibs ASD groups and ELGA groups.

## Results

### Word Production

Prior to analyzing differences in 18-month gesture production, we examined differences in 18-month word production to characterize the Sibs ASD and Sibs TD groups, and the ELGA and FT groups respectively.

Descriptive data on word production for Sibs TD, Sibs ASD-no LD, and Sibs ASD-LD are presented in Table 2. A significant difference in word production among the three groups was found (see Table 2). Specifically, Sibs ASD-LD produced significantly fewer words than Sibs ASD-no LD (*U* = 40; *p* ≤ 0.001) and Sibs TD (*U* = 19.5; *p* ≤ 0.001), but there was no difference between Sibs ASD-no LD and Sibs TD.

**TABLE 2:** Comparisons among Sibs TD, Sibs ASD-no LD and Sibs ASD-LD infants on gestures (rates per 10 min) and word production (CDI-WS) at 18 months (Kruskal-Wallis test).

	**Sibs TD (*n* = 29)**			**Sibs ASD-no LD (*n* = 28)**			**Sibs ASD-LD (*n* = 13)**				
**Gestures**	***M***	***SD***	***Range***	***M***	***SD***	***Range***	***M***	***SD***	***Range***	**χ*^2^***	***p***
Requesting/Reaching	0.26	0.43	0.00–2.00	0.09	0.23	0.00–0.80	0.21	0.57	0.00–1.98	5.68	0.06
Pointing	1.91	3.15	0.00–14.31	1.03	2.59	0.00–8.79	0.43	0.68	0.00–1.60	9.91	**0.01**
Showing	0.04	0.12	0.00–0.40	0.6	0.14	0.00–0.40	0.00	0.00	0.00–0.00	1.94	0.38
Giving	0.59	0.69	0.00–2.00	0.70	1.19	0.00–4.00	0.88	1.97	0.00–6.40	1.20	0.55
Conventional	0.26	0.46	0.00–1.60	0.16	0.49	0.00–2.40	0.06	0.15	0.00–0.40	2.60	0.27
Representational	0.00	0.00	0.00–0.00	0.00	0.00	0.00–0.00	0.00	0.00	0.00–0.00	0.00	1
Total gestures	3.07	3.69	0.00–17.10	2.03	3.03	0.00–10.80	1.59	2.18	0.00–6.80	5.90	0.05
**CDI-WS**											
Word production^∗^	107.58	97.62	11–354	90.96	92.67	3–347	17.08	11.41	2–33	21.12	**<0.001**

*Significant results are in bold (*p* < 0.05). ^∗^Data for 5 Sibs-TD and 1 Sibs ASD-no LD infants were missing.*

Descriptive data on word production for FT, ELGA-no LD and ELGA-LD infants are presented in Table 3. A significant difference in word production among the three groups was found (see Table 3). Specifically, ELGA-LD infants produced significantly fewer words in comparison to ELGA-no LD (*U* = 4; *p* = 0.001) and FT infants (*U* = 23; *p* = 0.001). In addition, even though the means of ELGA-no LD infants and FT infants fell within the normal range with respect to Italian normative values (Caselli et al., 2015), ELGA-no LD infants produced significantly more words than FT infants (*U* = 61.5; *p* = 0.044).

**TABLE 3:** Comparisons among FT, ELGA-no LD and ELGA-LD infants on gestures (rates per 10 min) and word production (CDI-WS) at 18 months (Kruskal-Wallis test).

	**FT (*n* = 20)**			**ELGA-no LD (*n* = 11)**			**ELGA-LD (*n* = 9)**				
**Gestures**	***M***	***SD***	***Range***	***M***	***SD***	***Range***	***M***	***SD***	***Range***	**χ*^2^***	***p***
Requesting/Reaching	0.58	0.68	0.00–2.92	0.81	0.50	0.00–1.51	0.84	1.04	0.00–3.12	2.63	0.27
Pointing	3.37	2.99	0.00–9.62	3.79	3.96	0.00–12.33	0.77	0.84	0.00–2.16	8.05	**0.018**
Showing	2.11	2.05	0.00–6.40	2.50	3.20	0.00–11.80	2.09	1.37	0.61–4.67	0.23	0.89
Giving	1.68	1.70	0.00–6.84	2.36	2.36	0.00–7.20	3.18	4.64	0.00–12.00	0.34	0.84
Conventional	0.96	0.99	0.00–4.00	0.71	1.12	0.00–3.67	0.22	0.34	0.00–0.86	5.57	0.06
Representational	0.15	0.33	0.00–1.31	0.09	0.21	0.00–0.61	0.00	0.00	0.00–0.00	2.55	0.28
Total gestures	8.84	4.08	2.96–16.41	10.27	6.88	4.40–28.42	7.09	4.82	2.75–16.67	2.05	0.36
**CDI-WS**											
Word production	34.55	28.26	5–108	61.27	38.87	8–109	6.89	4.51	1–16	16.32	**<0.001**

*Significant results are in bold (*p* < 0.05).*

### Gesture Production

Mean rates per 10 min of gestures produced by Sibs TD, Sibs ASD-no LD, and Sibs ASD-LD are presented in Table 2. Inspection of data revealed that for Sibs TD and Sibs ASD-no LD, the most frequently produced gesture was pointing, whereas giving was most frequent among the Sibs ASD-LD. In all three groups, showing, conventional, and representational gestures were very infrequent or absent.

**FIGURE 1:**
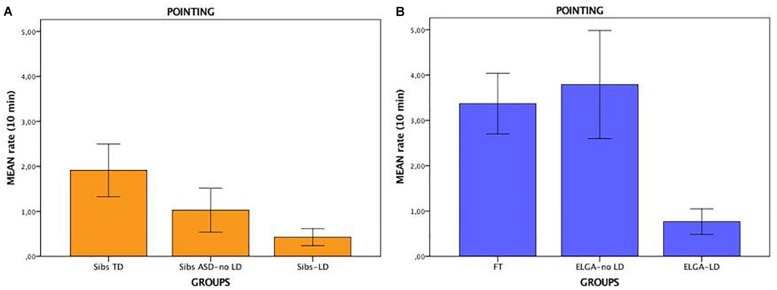
Mean rate of pointing for 10 min in Sibs TD, Sibs ASD-no LD and Sibs-LD infants **(A)** and in FT, ELGA-no LD and ELGA-LD infants **(B)**. Error bars represent ± 1 standard error.

Statistical analyses revealed a significant difference among the three groups in pointing gestures (see Table 2 and Figure 1). Follow-up Mann-Whitney tests indicated that Sibs ASD-LD (*U* = 116.5; *p* = 0.038) and Sibs ASD-no LD (*U* = 248; *p* = 0.004) produced significantly fewer pointing gestures compared to Sibs TD. Rate of pointing did not differ statistically between Sibs ASD-LD and Sibs ASD-no LD. No other comparisons reached statistical significance.

Mean rates per 10 min of gestures produced by FT, ELGA-no LD, and ELGA-LD infants are presented in Table 3. For the FT and ELGA- no LD groups, the most frequently produced gesture was pointing, but for the ELGA-LD, giving was most common. In all groups, representational gestures were very infrequent.

A significant difference in pointing gestures among the three groups was found (see Table 3 and Figure 1). Mann-Whitney tests revealed that ELGA-LD infants produced significantly fewer pointing gestures than ELGA-no LD (*U* = 20; *p* = 0.024) and FT infants (*U* = 32; *p* = 0.006). ELGA-no LD and FT infants did not differ statistically from one another. No other differences were statistically significant.

## Discussion

The present study was designed to examine gesture production at 18 months across two groups of infants with enhanced risk of language delay (LD), compared to their respective TD groups, to determine whether a common early index of risk exists among different populations at risk for LD. Our findings indicate that Sibs ASD with no eventual ASD diagnosis and ELGA infants without major cerebral damages, congenital malformations or sensory impairments who met criteria for LD between 18 and 36 months exhibited lower rates of pointing at 18 months during a naturalistic mother-infant interaction with respect to their Sibs TD and FT comparison groups. Lower rates of pointing at 18 months, though less pronounced, were found also in Sibs ASD with no LD with respect to their TD comparison group, whereas rate of pointing of ELGA infants with no LD did not differ significantly from their comparison FT group. Taken together these findings highlight the relevance of reduced rates of pointing at 18 months as an early common indicator of LD across different groups of infants. In addition, they highlight some differences between Sibs-ASD and ELGA infants concerning those infants who did not have a history of LD.

### Low Rates of Pointing in 18-Month-Old Sibs ASD and ELGA Infants as a Common Early Index of LD

Reduced rates of pointing were observed in the Sibs ASD-LD group at 18 months. This result extends previous work by LeBarton and Iverson (2016), who found that Sibs ASD-LD produced fewer pointing gestures at 24 and 36 months. Interestingly, even Sibs ASD-no LD exhibited a lower rate of pointing with respect to Sibs TD at 18 months. This is consistent with a study by Mitchell et al. (2006), who found delays in gesture production, assessed through the CDI, in Sibs ASD even when those with LD were excluded. In addition, a very recent study, measuring gestures with the CDI from 8 to 14 months, found lower gesture production in all Sibs ASD, with the lowest production observed in Sibs ASD later diagnosed with ASD, followed by Sibs ASD-LD and Sibs ASD-no LD (Iverson et al., 2018). Interestingly, a similar trend was observed in the current study, with Sibs ASD-LD exhibiting the lowest rate of pointing, whereas the difference in pointing rate between Sibs ASD-no LD and Sibs TD was less pronounced. Taken together, these findings suggest that the communicative-language domain may be particularly vulnerable in Sibs ASD even in absence of LD, and that a lower rate of pointing at 18 months is a common marker in these infants.

With regard to the preterm population, only one study specifically documented that ELGA infants pointed at a lower rate relative to FT infants at 12 months (Benassi et al., 2016) and, to date, no studies have investigated profiles of gesture production in ELGA infants in relation to language outcomes. Our study revealed that, compared to FT infants, a reduced production of pointing in ELGA infants persisted until at least 18 months of age for those with LD, whereas there were no differences observed for ELGA-no LD infants. Our findings thus highlight the existence of two different profiles within the ELGA population emerging in the second year of life, one with no LD (about 55%), the other with LD (about 45%) that can be identified around 18 months by a lower rate of pointing. ELGA-LD infants in the current study were characterized by more severe neonatal biological and medical characteristics (i.e., lower mean gestational age; longer hospitalization at birth) compared to ELGA-no LD peers. These findings suggest the hypothesis that severe neonatal biological and medical characteristics may contribute to the emergence of LD by affecting cortical maturation and, particularly, that of the temporal lobe and adjacent regions that are centers for language development. These neural structures are particularly vulnerable during the third trimester of gestation, a sensitive period for brain and body development that for preterm infants occurs in an artificial, frequently impoverished, and stressful environment (Sansavini et al., 2011a; Vohr, 2014). Indeed, decreased cortical volumes in several areas (sensorimotor, premotor, midtemporal, parietal, occipital) as well as altered microstructure and connectivity in the brains of preterm infants up to adolescence have been found in previous studies of infants born preterm, showing relations between structural and functional alterations in brain development and the emergence of neurodevelopmental disorders (Peterson et al., 2002; Mullen et al., 2011). Profiles of language development in ELGA infants merit further investigation in future studies in order to understand whether the reduced use of pointing is related to subtle alterations in brain development. Furthermore, it would be useful to study the developmental trajectory of gesture production at later ages in ELGA-LD infants as well as in less immature preterm infants with LD in order to examine whether a lower rate of pointing is common among preterm infants with LD and how rate and types of gesture production change in preterm infants with LD.

Our findings suggest thus that a lower rate of pointing at 18-month may be an early common marker of LD in Sibs ASD and ELGA infants. This is consistent with a recent study conducted on another group of infants at risk for LD, i.e., infants with a family history of LD, which revealed reduced use of pointing gestures at 12 and 14 months in children exhibiting LD at 24 months (Lüke et al., 2017). Lower production of communicative gestures between 18 and 28 months of age also distinguishes truly delayed late talkers from late bloomers, highlighting the predictive value of measures of gesture use for later expressive language skills (Thal and Tobias, 1992). Taken together, these findings underscore the relevance of analyzing use and rate of pointing in the second year as a potential index of later language acquisition/delay. Pointing at the beginning of the second year is related to the beginning of word comprehension and production, and it plays a key role in coordinating attention to persons, objects, and events with other people and to labels associated with them (Tomasello et al., 2007; Sansavini et al., 2010a). Thus, infants who point less frequently may have fewer opportunities to initiate and maintain joint attention with their caregivers and to associate labels with their referents in daily interactional contexts.

Our findings also suggest that, although Sibs ASD and ELGA infants present early common indicators of LD, they may differ in the nature and extent of their vulnerabilities in language. Indeed, Sibs ASD-no LD exhibited a lower rate of pointing than Sibs TD, whereas no significant differences were found between ELGA-no LD and FT infants. Thus, this cross-population study, conducted from early stages of development, contributed to shed light on the emergence of LD phenotypes in different populations at risk for LD, highlighting reduced rates of pointing at 18 months as an early reliable common marker of LD across Sibs ASD and ELGA infants, but with the former appearing more vulnerable in language development even in absence of LD outcome. Taken together, our results represent an initial step in the early identification of LD in Sibs ASD and ELGA infants, and they underscore the need for further work examining mechanisms underlying LD across different populations.

### Limitations

Some limitations that may impact the generalizability of our findings should be noted. First, gesture production was investigated at a single age point. Further longitudinal studies that include more than one age point in the second year of life are needed to understand similarities and differences in developmental trajectories of gesture use across different groups at risk for LD. Second, data were collected in the context of naturalistic mother-infant play interactions. This represented a strength of our research. However, we did not code parental input. As suggested by the literature, information conveyed in children’s gestures can influence the input that adults provide to children and this input can, in turn, support children’s learning during these interactions and promote language development (Goldin-Meadow et al., 2007; Tamis-LeMonda and Baumwell, 2011). Future work needs to examine caregivers’ input to infant gestures and, in particular, to pointing gestures across populations with LD to examine similarities and differences in parents’ communication to children. Third, the Sibs ASD-LD and ELGA-LD samples were both relatively small, since they specifically included only Sibs ASD with no ASD diagnosis and extremely preterm infants without major cerebral damages, congenital malformations and visual or hearing impairments. In order to understand the contribution, of biomedical risk factors for ELGA infants in predicting LD, future studies need to be conducted with a larger sample of ELGA infants. Finally, in our study we focused on two populations at risk for LD, compared to their TD groups. Further studies could be conducted to extend this comparison to a larger number of populations at risk for LD, including for instance infants with a family history of LD.

## Conclusion and Clinical Implications

This study demonstrates that a low rate of pointing gesture at 18 months may be a reliable and common marker of LD across different populations of infants with enhanced LD risk. This result has important clinical implications. First, this underscores the relevance of monitoring gesture production, especially pointing, during the second year that may be crucial for early identification of potential later LD.

Second, it broadens our understanding of the relations existing between gesture production and language delay, with relevant implications for interventions designed to support language development from its early stages, particularly in populations at risk for LD. Taking into account that reduced pointing production may shape the input that infants receive, which in turn may lead to cascading effects on subsequent language development, intervention must include parent coaching, in order to increase parents’ recognition of and enhance contingent responses to their infant’s gestures. Indeed, as revealed by some studies involving parents of Sibs ASD (Rogers et al., 2012; Kasari et al., 2014) and parents of preterm infants (Barrera et al., 1986; Spittle et al., 2015), parent coaching interventions improve parent sensitivity to children’s communication bids and parent responsiveness. These in turn may lead to significant gains in children’s language skills over time. This kind of intervention may create the basis for an enriched environment that could positively affect child language development, especially for at risk populations such as Sibs ASD and infants born preterm. Parent coaching for parents of Sibs ASD and ELGA infants with a low rate of pointing gesture at 18 months should thus be studied with randomized controlled trials to evaluate their effectiveness in supporting language development from its early stages.

## Data Availability

The datasets for this manuscript are not publicly available because they include sensitive data of children. Data are kept in the lab by the authors of the paper. Requests to access the datasets should be directed to alessandra.sansavini@unibo.it; jiverson@pitt.edu.

## Ethics Statement

All study procedures met the ethical guidelines for protection of human participants, including adherence to the legal requirements of the Country, and received a formal approval by the local Ethical Committees. The study from which Sibs ASD and Sibs TD were drawn was approved by the University of Pittsburgh Institutional Review Board. The ELGA and FT infant study was approved by the Ethical Committee of the Hospital of University of Bologna. The parents of all infants provided written informed consent for participation in the study, data analysis, and anonymized data publication.

## Author Contributions

AS, JMI, and AG designed and conceptualized the study, methods, data collection, coding, and analyses. GF supervised the medical data collection of the preterm sample and medical aspects of the method. JZL and MZ coded the data. AG, MZ, JZL, and AS analyzed the data. AS, MZ, and AG wrote the manuscript. JMI, AS, and AG reviewed the manuscript. All authors approved the final manuscript.

## Conflict of Interest Statement

The authors declare that the research was conducted in the absence of any commercial or financial relationships that could be construed as a potential conflict of interest.
