# Supplementary material for: Low Rates of Pointing in 18-Month-Olds at Risk for Autism Spectrum Disorder and Extremely Preterm Infants: A Common Index of Language Delay?
Source: Front Psychol. 2019 Oct 9;10:2131. doi: 10.3389/fpsyg.2019.02131 (PMC6794419; doi:10.3389/fpsyg.2019.02131)
Supplement: Supplementary file 1 [file Table_1.pdf]

Supplementary File.

Biological and medical characteristics of ELGA infants and comparison of biological and medical characteristics between the ELGA-no LD and the ELGA-LD groups.

Significant results are in bold (p < .05).

	ELGA (n = 20)	ELGA-no LD (n = 11)	ELGA-LD (n = 9)	t/χ^2	p
Weeks of gestational age, *M (DS) range*	25.8 (1.5) 23-28	26.5 (1.2) 25-28	24.9 (1.3) 23-26	.99	**.012**
Birthweight, *M (DS) range*	803 (191) 509-1093	862 (172) 516-1093	730 (198) 509-1040	.43	.128
Hospitalization days, *M (DS) range*	91.5 (32.2) 49-152	73.5 (22.9) 49-113	113.4 (28.5) 76-152	.24	**.003**
Caesarean section, *n* (%)	17 (85)	10 (91)	7 (78)	1.82	.285
First born, *n* (%)	15 (75)	9 (82)	6 (67)	.61	.617
Later born, *n* (%)	5 (25)	2 (18)	3 (33)	.61	.617
Multiple births, *n* (%)	6 (30)	3 (27)	3 (33)	.64	.642
BW < 1000 g, *n* (%)	16 (80)	8 (73)	8 (89)	.81	.369
SGA, *n* (%)	2 (10)	1 (9)	1 (11)	.022	1
MV, *n* (%)	11 (55)	5 (46)	6 (67)	.90	.406
RDS, *n* (%)	20 (100)	11 (100)	9 (100)	-	
Apnoea, *n* (%)	6 (30)	4 (36)	2 (22)	.47	.426
BPD, *n* (%)	12 (60)	5 (46)	8 (89)	4.10	.070
IVH I/II, *n* (%)	1 (5)	0 (0)	1 (11)	1.29	.450
HE (≥14d.), *n* (%)	17 (85)	10 (91)	7 (78)	1.34	.511
ROP I/II, *n* (%)	13 (65)	6 (56)	7 (78)	1.80	.406
Sepsis, *n* (%)	6 (30)	1 (9)	5 (56)	5.09	.050
Hyperbil.with phototer., *n* (%)	16 (80)	8 (73)	8 (89)	1.30	5.22

Medical complications (infants could have one or more medical complications): MV: mechanical ventilation. **SGA**: infants with a birthweight <10th percentile for gestational age. **RDS**: respiratory distress syndrome, acute illness coming on within 4-6 h of delivery, clinically characterized by a respiratory rate ≥60/min, dysponea and respiratory distress. **Apnoea**: significant apnoea was defined as more than four episodes of apnoea/hour or more than two episodes of apnoea/hour if ventilation with bag and mask was required. **BPD**: bronchopulmonary dysplasia,

need of both supplemental oxygen for ≥28 days and at 36 weeks of post-conceptional age. **IVH I/II**: intra-ventricular haemorrhage originating within the subependymal germinal matrix filling less than respectively 10% (I grade) and 50% (II grade) of the ventricular area on parasagittal view. **HE**: hyperecogenicity, a prolonged flare ≥14 days without cystic lesions and/or ventricular dilatation and that resolved completely without any abnormality in its place. **ROP I/II**: retinopathy of prematurity, vasoproliferative retinopathy that resolved without a specific therapy before the presumed date of birth. **Sepsis**: presence of a positive blood culture and/or clinical and laboratoristic signs. **Hyperbil. with phototer**.: hyperbilirubinemia needing phototherapy according to the criteria proposed by Gomella (2009).
